# Capabilities, opportunities and motivations of staff to provide hearing support to long-term care home residents with dementia

**DOI:** 10.1080/14992027.2023.2227764

**Published:** 2023-06-29

**Authors:** Hannah Cross, Christopher J. Armitage, Piers Dawes, Iracema Leroi, Rebecca E. Millman

**Affiliations:** a Manchester Centre for Audiology and Deafness, School of Health Sciences, University of Manchester, Manchester, UK; b Manchester Centre for Health Psychology, University of Manchester, Manchester, UK; c Manchester University NHS Foundation Trust, Manchester Academic Health Science Centre, Manchester, UK; d NIHR Greater Manchester Patient Safety Translational Research Centre, University of Manchester, Manchester, UK; e NIHR Manchester Biomedical Research Centre, Manchester University Hospitals NHS Foundation Trust, Manchester Academic Health Science Centre, Manchester, UK; f Centre for Hearing Research (CHEAR), School of Health and Rehabilitation Sciences, University of Queensland, Saint Lucia, Australia; g Global Brain Health Institute and School of Medicine, Trinity College Dublin, Dublin, Ireland

**Keywords:** Behaviour Change Wheel, COM-B model, Residential Care, Dementia Care, hearing loss

## Abstract

**Objective:**

Many long-term care home (LTCH) residents have dementia and hearing loss, causing communication difficulties and agitation. Residents rely on staff for hearing support, but provision is often inconsistent. This study used the Behaviour Change Wheel’s Capability, Opportunity and Motivation model to understand why LTCH staff do or do not, provide hearing support to residents with dementia who they believe could benefit from it.

**Design:**

An online survey exploring hearing support provision, capabilities, opportunities, motivations and demographics. Data were analysed using descriptive statistics, within-participants ANOVA and multiple linear regression.

**Study sample:**

165 LTCH staff.

**Results:**

Staff provided hearing support to 50% of residents with dementia who they thought would benefit. Self-reported physical and psychological capabilities (skills/knowledge) were significantly higher than physical opportunity (having time/resources). The physical capability was significantly higher than social opportunity (collaborative working) and reflective motivation (feeling motivated). Lower levels of hearing support provision were predicted by LTCH funding (private vs. local authority), job role (care assistant vs. nurse) and fewer physical opportunities.

**Conclusions:**

Boosting capabilities through training alone may not be as effective as increasing opportunities via environmental restructuring. Opportunities may include strengthening working relationships with audiologists and ensuring hearing and communication aids are available within LTCHs.

## Introduction

At least 70% of long-term care home (LTCH) residents have dementia (Prince et al. 2014) and 75% have hearing loss (Royal National Institute for Deaf People, 2018). Many residents are negatively affected by the overlapping and interacting symptoms of these conditions: Untreated and under-treated hearing loss can exacerbate agitation (Haque, Abdelrehman, and Alavi 2012), communication difficulties (Crosbie et al. 2019), loneliness, activity limitations and poorer quality-of-life (Punch and Horstmanshof 2019). Providing hearing support to residents with dementia, including hearing aids or other amplification devices, visual aids and utilising communication techniques can improve outcomes for residents and reduce staff “burden” and stress (see Cross et al. (2022) for a systematic review).

Supporting residents’ hearing needs is essential. However, recent evidence emphasises the need for improved hearing support within LTCHs: Staff training in hearing is lacking and hearing is of low priority (Cross et al. 2022; Leroi et al. 2021; White et al. 2021). Many residents with dementia rely on caregivers to meet their hearing-related needs (White et al. 2021). Therefore, LTCH staff must be equipped to provide this critical support.

Understanding who would benefit, and in which domain/area, is a necessary first step in the development of behaviour change interventions to support staff in providing hearing support. Previous studies have considered LTCH staff work-related demographic factors, e.g. job title, years of experience and LTCH funding and registration type. However, results have been conflicting. For example, having fewer years of experience working in LTCHs has been associated with uncertainty about hearing practices in their place of work (Andrusjak, Barbosa, and Mountain 2021) and poorer capacity to support the hearing needs of residents (Dawes et al. 2021), but not with knowledge of hearing support (Andrusjak, Barbosa, and Mountain 2021).

Job title (working as a care assistant) and type of LTCH registration (non-dementia specialist) was associated with uncertainty about hearing support practices in one study (Andrusjak, Barbosa, and Mountain 2021), but did not impact results in another (Leroi et al. 2021). Furthermore, LTCH funding type (privately owned) predicted poorer capacity to support residents’ hearing needs (Leroi et al. 2021), but this effect was not replicated in similar surveys (Andrusjak, Barbosa, and Mountain 2021, Dawes et al. 2021). Finally, LTCH size (smaller homes) predicted greater capacity to provide hearing support (Leroi et al. 2021), but had no impact on hearing practices reported elsewhere (Andrusjak, Barbosa, and Mountain 2021). Therefore, further investigation into whether these demographics predict the provision of hearing support is required, for the first time using a distinct “behaviour” measure, so that behaviour change interventions can target the necessary professional groups.

In addition to demographic predictors, an understanding of the psychosocial drivers that prohibit behaviour (hearing support provision) is required. The Behaviour Change Wheel (BCW; Michie, Van Stralen, and West 2011) allows for evidence-based intervention development and implementation. At the hub of the BCW is the Capability, Opportunity, Motivation model of Behaviour (COM-B), which can be used to gain an understanding of what needs to change to bring about effective behaviour change. According to COM-B, behaviour is driven by people’s physical capability (e.g. skills), psychological capability (e.g. knowledge), physical opportunity (e.g. time), social opportunity (e.g. systemic issues), reflective motivation (e.g. goals) and automatic motivation (e.g. habits). The BCW can aid in selecting relevant intervention functions and policy categories to elicit behaviour change, based on the domain(s) identified as lacking.

Previous literature has not considered COM-B in relation to hearing support in LTCHs. However, elements of the model have been tested in isolation. For example, previous studies show that LTCH staff knowledge (psychological capability) of hearing loss is variable, and training on hearing aid management skills (physical capability) is non-mandatory (McShea and Ferguson 2022; Solheim, Shiryaeva, and Kvaerner 2016). Alternatively, it has been suggested that opportunities for LTCHs to access external audiology services are fragmented (White et al. 2021), hearing-related resources are unavailable within LCTHs (Bott et al. 2022) and LTCHs are unsuitable environments for communicating with residents with hearing loss (Pryce and Gooberman-Hill, 2012). Investigations into staff motivation to provide hearing support have, to our knowledge, not been investigated.

This study will, for the first time, use validated measures of the COM-B model to identify the predictors of LTCH staffs’ provision of hearing support to residents with dementia that they think would benefit. These results will aid in the development of a behaviour change intervention (Michie, Van Stralen, and West 2011) for staff to improve hearing support in LTCHs, by understanding which staff, and in which domains, interventions should target.

### Research questions

1. What proportion of residents with dementia that LTCH staff think would benefit from hearing support receive it?
2. What are the capabilities, opportunities and motivations of LTCH staff to provide hearing support to LTCH residents with dementia that they think would benefit?
3. Do the capabilities, opportunities, motivations and work-related demographic factors of LTCH staff predict the provision of hearing support to residents with dementia that they think would benefit?”

## Materials and methods

### Study design

A UK-based online study was undertaken. The study was pre-registered on the Open Science Framework (https://osf.io/t2whm/). Study data were collected and managed using REDCap (Harris et al. 2009) between October 2020 and July 2021. Respondents provided written informed consent using an online consent form before beginning the survey and were remunerated with a £5 High Street e-Voucher. The study received ethical approval from the University of Manchester Research Ethics Committee (ref: 2020-10261-16439).

### Participants

LTCHs are residential settings where several older people live and have access to 24-hour care. LTCHs include Residential Care Homes which provide accommodation, meals and personal care, Care Homes with Nursing employ registered nurses for complex health needs, and Dementia Specialist Homes support residents with advanced cognitive and behavioural needs. Staff aged over 16 years who were working in any of these LTCHs were eligible to participate, providing that their place of work supported residents with dementia. The study was open to staff involved in direct care. People working in LTCHs who do not provide care (e.g. domestic, kitchen staff) were excluded from participating.

Participants were recruited via convenience sampling, which was deemed appropriate given the anticipated challenges of recruiting LTCH staff during COVID-19 lockdowns. Participants were recruited through email advertisements distributed to LTCH managers in the National Institute for Health and Social Care Research’s (NIHR) ENabling Research in Care Homes (ENRICH) Network (https://enrich.nihr.ac.uk/). Managers received a study recruitment poster containing the survey link and were encouraged to distribute this poster to all care-related LTCH staff under their management. To aid recruitment, an advert was placed in the Care Home Management Magazine (https://chmonline.co.uk/), posted on social media platforms and shared via word of mouth.

### Measures

The survey can be found in the Supplementary Materials.

Pilot testing of a draft survey with five LTCH staff (one nurse, three care assistants and one occupational therapy assistant) working across England and Wales revealed that the survey was easy to access, understand and no modifications were required.

Participants’ sociodemographic and work-related demographic measures were collected.

To understand behaviour, participants were asked to respond on 0-100% visual scales for the following three statements: “*Out of the residents that you care for, how many have dementia?”,* “*Out of the residents* ***with dementia*** *that you care for, how many do you think would benefit from hearing loss support?*” and *“Out of the residents* ***with dementia that you think would benefit****, how many do you provide hearing loss support to?”* The latter item was used to operationalise the target behaviour. “Hearing loss support” was defined as “*helping residents with their hearing aids or other hearing devices, using communication aides such as pictures or flashcards or changing your communication techniques to help those with hearing loss”* to encompass the multiple methods used in LTCHs (Cross et al. 2022).

The items included in the survey were developed based on a brief validated COM-B measure (Keyworth et al. 2020), which requires respondents to report their perceptions of physical capability, psychological capability, physical opportunity, social opportunity, reflective motivation and automatic motivation. Participants responded on 11-point scales (0 Strongly Disagree − 10 Strongly Agree) for each sub-domain. For example, the physical capability was assessed by *“I am* ***physically*** *able to provide hearing loss support for residents with dementia”*, accompanied by a short definition of the sub-domain within the context *“For example: having the skills to insert hearing aids or change batteries.”*

Additional questions further explored the provision of hearing support, e.g. access to training and staff delegation, which include a range of Yes/No, Likert scale and open-ended questions.

### Analyses

Data were exported from REDCap into IBM SPSS V.25 for analysis.

Descriptive statistics were used to summarise respondent demographics, COM-B items and additional quantitative items. Optional open-ended qualitative responses were analysed using inductive manifest content analysis (Hsieh and Shannon 2005), which involved identifying and quantifying codes for each question to further expand on quantitative questions. An additional exploratory chi-squared analysis was used to assess the relationship between receiving training on hearing devices and testing and checking hearing aids.

A within-participants ANOVA was used to evaluate differences between individuals’ self-reports of COM. Visual inspection of boxplots revealed no significantly influencing outliers and Normal Quantile-Quantile plots showed that the data were distributed normally. The assumption of sphericity was violated (Mauchly’s Test, *p* < 0.001). Therefore, Huynh-Feldt correction was applied (ε = 0.93). Bonferroni adjustments for multiple comparisons were applied for post-hoc analyses. Two participants did not provide a response to the physical opportunity item, and so were excluded from this analysis (*N* = 163 in the ANOVA).

A multiple linear regression model was used to explore the relationship between the provision of hearing loss support by LTCH staff to residents with dementia (behaviour) and five demographic factors that could potentially influence behaviour (job title, years of experience, LTCH type, LTCH funding, number of resident bedrooms) and the six COM sub-domains. Five respondents did not report their job title, place of work, funding type or size of LTCH, and were therefore excluded from the regression analysis. Six influential outliers were identified via boxplot inspection (3 for years in profession, 3 for the number of resident bedrooms in LTCH) and removed (*N* = 152 in the multiple linear regression).

The data used in the regression model met the following assumptions: linear relationships between the dependent variable and independent continuous variables (assessed using scatterplot inspection), collinearity (all Tolerance scores >0.1, all Variance Inflation Factors <10; Field, 2013) and independent errors (Durbin–Watson statistic *d* = 1.8). Multivariate normality was confirmed using Quantile-Quantile Plots. Visual inspection of the histograms and Probability-Probability plots of standardised residuals revealed normally distributed values. Visual inspection of scatterplots of standardised residuals showed that assumptions of homogeneity of variance were also met.

Categorical variables (job role, LTCH type and LTCH funding) were recoded into *k −* 1 dummy variables, where *k* is the number of levels in the original variable. Reference variables were Care Assistant (for job title), Dementia Specialist Home (for LTCH type) and Local Authority (for LTCH funding).

An alpha level of *α* ≤ 0.05 was used to determine the statistical significance of the regression results.

### Sample size

An *a priori* power calculation (G*Power; Faul et al. 2009) revealed that a sample size of *N* = 137 participants was required to obtain a medium effect size (*f* = 0.39) with an estimated power of 80% in a multiple linear regression model with 15 predictors (LTCH type (2 dummy variables), LTCH funding (1 dummy variable), job role (4 dummy variables), number of resident bedrooms in LTCH, years in profession, physical capability, psychological capability, physical opportunity, social opportunity, reflective motivation, automatic motivation).

## Results

### Participant characteristics

Participant demographics are presented in Table 1. Most respondents were white (95.2%) and female (75.8%) and 52.1% were educated to degree level or equivalent. The mean age of respondents was 38.6 years (*SD* = 8.2, range: 19–64 years).

**Table 1.** Participant Demographics.

Variable	*N* (%)	Mean (*SD*)
Gender		
Female	125 (75.8)	
Male	40 (24.2)	
Age (years)		38.6 (8.2)
Ethnicity		
White	157 (95.2)	
Asian/Asian British	5 (3.0)	
Mixed/Multiple ethnic groups	1 (0.6)	
Any other ethnic group	1 (0.6)	
Prefer not to answer	1 (0.6)	
Highest level of UK educational qualification^a^		
No qualifications	2 (1.2)	
GCSE or equivalent	9 (5.5)	
A-Level or equivalent	19 (11.5)	
Diploma	35 (21.2)	
Bachelors/Undergraduate degree or equivalent	86 (52.1)	
Postgraduate degree	7 (4.2)	
Other	5 (3.0)	
Prefer not to say	2 (1.2)	
LTCH type^b^		
Care Home with Nursing	132 (80.0)	
Residential Care Home	23 (13.9)	
Dementia Specialist Home	8 (4.8)	
LTCH funding type^c^		
Private company	140 (84.8)	
Local authority	24 (14.5)	
Job title		
Care assistant	90 (54.5)	
‘Other’	10 (6.1)	
Senior carer	31 (18.8)	
Registered nurse	28 (17.0)	
LTCH manager	5 (3.0)	
Number of resident bedrooms in LTCH		65.8 (33.5)
Fewer than 21 (Small)	10 (6.2)	
21–40 (Medium)	17 (10.6)	
40 or more (Large)	134 (83.2)	
Years in profession		10.4 (6.4)
3 or fewer	23 (14.2)	
4–9	58 (35.8)	
10 or more	78 (50.0)	

^a^GCSE: academic qualifications taken in UK full-time education, usually at 16 years old. A-Level: academic qualifications taken in UK full-time education, usually at 18 years old.

^b^Residential Care Home: accommodation, meals, personal care and support provided. Nursing Home (or Care Home with Nursing): registered nurses for residents with complex health needs also employed. Dementia Specialist Homes: dementia care for residents with advanced cognitive and behavioural needs.

^c^Local Authority funded: LTCHs owned by the UK local district, borough or county council.

Respondents included care assistants (54.5%), senior carers (18.8%), registered nurses (17.0%), managers (3.0%) and “other” (6.1%). The mean number of years working in the care profession was 10.4 (*SD* = 6.4; range: 1–35 years). Most respondents reported working in Care Homes with Nursing (80.0%), followed by Residential Care Homes (13.9%), and Dementia-Specialist Homes (4.8%). Most LTCHs were large, with 40+ bedrooms (83.2%, range: 2–180 bedrooms).

### Behaviour: providing hearing support to residents with dementia

Respondents reported that, on average, 54.2% (*SD* = 24.9, range: 14–100%) of the residents that they care for have dementia. Out of these residents with dementia, they believed that 48.5% (*SD* = 20.3, range: 6–100%) would benefit from hearing loss support. However, LTCH staff reported providing hearing support to only 50.0% *(SD* = 20.7, range: 0–100%) of those who they thought would benefit, indicating that half of the residents with dementia and hearing loss do not receive hearing loss support from LTCH staff.

### Capability, opportunity and motivation to provide hearing support

A within-participants ANOVA (with Huynh-Feldt correction) revealed significant differences between LTCH staffs’ individual self-reports of COM, *F*(4.50, 728.37) = 6.35, *p* < .001. Post-hoc analysis (Bonferroni correction applied), showed that physical capability scores (*Mean* = 7.73, *SD* = 2.20) were significantly higher than those of reflective motivation (*Mean* = 7.07, *SD* = 2.34) (*p* = 0.002), physical opportunity (*Mean* = 6.87, *SD* = 2.22) (*p* < .001) and social opportunity (*Mean* = 6.98, *SD* = 2.29) (*p* = 0.002). Psychological capability scores (*Mean* = 7.44, *SD* = 1.97) were also significantly higher than physical opportunity scores (*Mean* = 6.87, *SD* = 2.22) (*p* = .013). Automatic motivation scores (*Mean* = 7.15, *SD* = 2.24) did not differ from any other domain.

### Predictors of providing hearing support to residents with dementia (behaviour)

Table 2 shows the results of the multiple linear regression analysis used to assess predictors of providing hearing support to residents with dementia that staff thought would benefit. A significant effect of the predictors on the target behaviour was found, *F*(15,142) = 3.04, *p* < .001, and can be further understood by examining the associations between behaviour and predictors entered into the regression model (Supplementary Table).

**Table 2.** Linear model predictors (Job title, years of experience, LTCH type, LTCH funding, number of resident bedrooms in LTCH and COM domains) of behaviour (providing hearing support to residents with dementia).

Variable	*B*	SE B	*β*	*t*	95% CI	*p*
Constant	39.24	12.56		3.12	14.41, 64.07	**.002**
LTCH type:						
LTCH nursing	4.67	7.76	.09	0.60	−10.66, 20.00	.548
LTCH residential	9.09	8.35	.15	1.09	−7.41, 25.59	.278
LTCH funding:						
Private company	−14.14	4.99	−.24	−2.83	−24.00, −4.28	.**005**
Job role:						
‘Other’ role	13.17	7.48	.15	1.76	−1.61, 27.96	.080
Senior carer	7.38	4.88	.14	1.52	−2.25, 17.02	.132
Registered nurse	17.332	4.84	.32	3.58	7.75, 26.91	**.000**
Manager	2.11	10.10	.02	0.21	−17.86, 22.09	.835
Number of bedrooms	0.03	0.06	.04	0.43	−0.10, 0.15	.666
Years in profession	−0.52	0.26	−0.16	−1.97	−1.04, 0.00	.051
COM domains:						
Physical capability	−1.91	1.03	−.20	−1.86	−3.99, 0.12	.065
Psychological capability	0.79	1.19	.08	0.66	−1.57, 3.15	.508
Automatic motivation	1.51	0.86	.16	1.76	−0.18, 3.20	.080
Reflective motivation	0.56	0.97	.06	0.58	−1.36, 2.47	.566
Physical opportunity	2.14	1.01	.23	2.12	0.14, 4.15	**.036**
Social opportunity	−0.63	0.97	−.07	−0.65	−2.55, 1.29	.517

*R*^2^ = 0.243, adjusted *R*^2^ = 0.163.

Behaviour was predicted by LTCH funding type (*p* = .005): Staff working in privately owned homes reported providing hearing support to fewer residents with dementia that they thought would benefit (*Mean* = 49.1, *SD* = 20.2) than those working in local authority homes (*Mean* = 57.5, *SD* = 20.5). The job title was also a significant predictor of behaviour (*p* < .000): Registered nurses reported providing hearing support to more residents with dementia that they thought would benefit (*Mean* = 59.9, *SD* = 26.2) compared to care assistants, who reported providing support to the fewest residents (*Mean* = 44.8, *SD* = 17.9). LTCH type, number of resident bedrooms and years of experience were not significant predictors. Physical opportunity significantly predicted behaviour (*p* = .036): those who perceived themselves to have the greater physical opportunity provided hearing support to more residents with dementia that they thought would benefit.

### Additional barriers to providing hearing support

Only 26.7% of respondents (*N* = 165) reported testing or checking hearing aids. Open-ended responses to methods used centred around checking batteries or listening for whistling noises. Only 24.8% reported having had any training and support on hearing devices, and 83.6% reported wanting more training in this area, mainly on hearing aid management “*I would like to know more about the battery’s (sic) etc.*”, “*how to look after hearing aids, how to test them, clean them*” and empathy training “*what it’s like to have hearing loss*”. There was a significant relationship between receiving training on hearing devices and testing and checking residents’ hearing aids χ(1) = 21.990, *p* < .001.

In terms of perceived responsibilities for providing hearing support, 46.1% of respondents believed care assistants to be most responsible, followed by registered nurses (33.3%). Only 14.5% thought provision is a collaborative responsibility. LTCH staff did not regard relatives (1.8%) or the residents themselves (1.8%) to be responsible for hearing support. Only 30.9% had a specifically designated staff member responsible for hearing support in their place of work. When asked whether hearing loss was a high priority compared to other care needs, 68.4% responded ≥7 on a 0–10 scale (*Mean* = 7.2, *SD* = 2.2).

When asked whether hearing support should be adapted for residents with dementia, 72.1% of LTCH staff responded ≥7 on a 0–10 scale (*Mean* = 7.5, *SD* = 2.3). Open-ended responses focussed on difficulties with hearing aids: “*Not tolerating their aids, taking them out and hiding them sometimes. may be uncomfortable for them.*”, “*People with a dementia do not tolerate wearing objects that do not fit comfortably/cause irritation*”. Responses also highlighted that residents did not understand their hearing needs “*People with dementia do not always understand the need to use their hearing aids and being able to have the extra time to support and explain to them why it is important would be a great help*.”

When asked whether most residents with dementia use a hearing device efficiently, 64.3% of respondents scored ≥7; *Mean* = 6.7, *SD* = 2.3). For respondents who provided answers as to why not, “*not tolerated/refuses*” (27.9%) was the most frequent response. Other reasons included “*lost or broken*” (9.7%), “*hard to use*” (5.5%), “*resident forgets to use them*” (2.6%), “*not fitting well*” (2.4%), “*too expensive*” (1.2%) and “*not effective*” (0.6%).

## Discussion

This study aimed to understand why LTCH staff do or do not, provide hearing support to residents with dementia who they thought would benefit. On average, only half of residents with dementia who staff believed would benefit from hearing support, received this. Fewer than 25% of LTCH staff reported testing or checking residents’ hearing aids.

Identification of target COM-B domains and work-related demographics, which predict Behaviour is the first stage of developing hearing-related behaviour change interventions for LTCHs. Current results suggest physical opportunities of care assistants and those working in private LTCHs would be best targeted in future interventions, as opposed to focussing on capabilities alone.

### The influence of work-related demographics

Self-reported provision of hearing support was significantly lower for care assistants (responsible for assisting residents with personal care, meals, mobility etc.) compared to registered nurses (responsible for administering medication, providing more advanced nursing care and care planning). This is concerning as respondents also regarded care assistants as the members of staff who are most responsible for hearing support. These findings are consistent with Andrusjak, Barbosa, and Mountain (2021) where care assistants were more unsure of access to hearing screening tools, devices and assessments within their place of work than managers and nurses. However, results contrast with those of Leroi et al. (2021), in which job title appeared to be unrelated to knowledge, attitudes, or practices regarding hearing support. This discrepancy is likely attributable to the fact that the effects of job role were not formally analysed in Leroi et al. (2021).

LTCH staff working in privately owned homes also provided less hearing support to residents with dementia that they thought would benefit than those working in local authority-owned homes, consistent with findings that privately funded LTCHs have less capacity to support residents’ hearing loss (Leroi et al. 2021). The current study is the first to attribute LTCH funding type to the provision of hearing support using a distinct behaviour measure. Privately owned “profit driven” LTCHs are the most common type in the UK (Blakeley and Quilter-Pinner, 2019) and typically provide poorer care, have fewer resources and lower staffing levels (Winblad, Blomqvist, and Karlsson 2017), likely impacting hearing support. In private care settings, it may be that care deemed most essential is prioritised over psychosocial and communication-based support, as discussed in Ludlow et al. (2020).

Care assistants and those working in private LTCHs would benefit most from behaviour-change interventions to better equip them to provide hearing support to residents with dementia. As 84% of UK LTCH staff work as direct care assistants and 79% of filled posts in UK social care are independent (private) sector employers (Skills for Care, 2022), these staff are priorities for intervention.

### The influence of capabilities, opportunities, and motivation

#### Capability

In contrast with previous literature attributing poor hearing practices solely to a lack of staff knowledge of hearing loss and hearing aids (Kwak et al. 2022; Solheim, Shiryaeva, and Kvaerner 2016), LTCH staff in the current study rated their physical and psychological capability significantly higher than other domains, suggesting that they viewed their capabilities as less of a barrier. Despite feeling generally competent in the knowledge and skills required to provide hearing support to residents, most respondents had never received training in this area, mirroring the results of prior surveys (Cohen-Mansfield and Taylor 2004; Norwood-Chapman and Burchfield 2000). It may be that hearing device management is something currently learnt “on the job” through experience and modelling (having an example to imitate) other staff members. Nevertheless, it is important for LTCH staff to receive training and education about hearing loss. Our exploratory chi squared analysis revealed a significant relationship between receiving training on hearing devices and checking hearing devices. However, implementation of knowledge and skills may only be effective in the appropriate environmental context with sufficient opportunities, which is something that participants in the current study reported lacking.

#### Opportunity

Of all the COM sub-domains, respondents scored lowest on the physical opportunity (e.g. time, resources, funds) and social opportunity (e.g. working alongside other staff). This finding of limited access to opportunities is consistent with previous work recognising time pressures (Cross et al. 2022) and the unavailability of hearing and communication aids for residents with hearing loss (Bott et al. 2022; White et al. 2021) as barriers to hearing support in LTCHs.

Opportunities for LTCH staff to work alongside audiology services can also be an issue, as discussed in-depth in Cross et al. (under review). For example, difficulties obtaining accessible appointments that take place in LTCHs for residents with more advanced dementia. This fragmented working relationship limits opportunities for residents to undergo hearing assessments, thus changes in hearing may go unnoticed for residents unable to communicate or realise their own hearing loss (Andrusjak, Barbosa, and Mountain 2021; White et al. 2021). Furthermore, lengthy waiting lists may leave residents without appropriate hearing devices (Looi et al. 2004). Further investigation into the specifics of both social and physical opportunities for this critical collaboration between LTCHs and audiologists is required to optimise the effectiveness of interventions and bring benefit to both sectors.

#### Motivation

Respondents were significantly less motivated than they were physically capable to provide hearing support. As motivation is influenced by capability and opportunity (Michie, Van Stralen, and West 2011), scarce opportunities may result in LTCH staff believing that the provision of hearing support without the physical environment required to do so is too effortful. Furthermore, the negative impact of COVID-19 on the mental health of care assistants (Brady et al. 2022), may have affected work-related motivation, including provision of hearing support. Motivation can also be influenced by the behaviour itself (Michie, Van Stralen, and West 2011): Staff may enter a negative feedback loop of not supporting residents’ hearing. More specifically, motivation may be driven by residents’ responses to receiving hearing support: Refusal or rejection of hearing aids was the most reported reason for non-use in this study, consistent with the removal, physical discomfort, misplacement, breaking and inappropriate use reported in Jupiter (2016) and Leroi et al. (2021). These barriers likely decrease staff motivation to provide support, particularly where the benefits of hearing support may not be obvious.

### Developing interventions to improve physical opportunity

The physical opportunity was the only significant COM domain predictor of behaviour, and therefore a priority for intervention. BCW Intervention Functions relating to physical opportunity include “restriction” (rules to reduce engagement with competing behaviours), “environmental restructuring” (changing the physical environment), and “enablement” (increasing the means/reducing barriers to increase opportunity, not covered by other intervention functions) (Michie, Van Stralen, and West 2011). Restriction is not appropriate in this context, as introducing rules to reduce engagement with competing behaviours may, in turn, reduce the provision of other important care. Environmental restructuring could include reminders in resident care plans to insert/check hearing devices. Ensuring that there is an adequate supply of hearing aid batteries or visual aids within homes would also boost opportunities to provide hearing and communication support. Furthermore, enablement could involve strengthening interdisciplinary relationships (both physical and social opportunities) between audiology services and LTCH staff, so that hearing aids and other listening devices can be accessed, maintained and replaced more easily. Opportunity-based interventions that make the provision of hearing support physically easier for staff are also likely to improve LTCH staff opportunities, thus increasing target behaviour.

Increasing staff time, resources and funds (physical opportunities) in social care are larger issues that require systemic changes. Staff workload and time pressures are ongoing issues (Hayes, Tarrant, and Walters 2020; Skills for Care, 2022), and impacts good-quality resident-centred care (McGilton et al. 2014). Employing more LTCH staff to distribute workload, or workers specifically responsible for sensory care, is desirable but not practical in the short-term where there is a national shortage of LTCH staff (Skills for Care, 2022). Efforts to resolve this issue must be ongoing, alongside smaller-scale hearing interventions. Interventions should ideally be co-developed with LTCH staff, family and residents to determine what is feasible and has the best chance of success within LTCH settings using the APEASE (Acceptability, Practicability, Effectiveness, Affordability, Side-effects, and Equity) criteria (Michie, Van Stralen, and West 2011).

### Strengths and limitations

The use of self-report methodology introduces bias. Respondents may have over-estimated or over-reported their COM, particularly their perceived capabilities, in providing hearing support to affirm their identity as a caregiver with abilities to provide adequate care to residents (Brenner and DeLamater 2016). It is possible that staff may not even be aware that they lack knowledge and skills surrounding hearing loss. Using observational methods in future studies could provide a more reliable picture of the true capabilities, opportunities and motivations of LTCH staff, which could then be interpreted in conjunction with survey responses.

Furthermore, there are limitations to the “behaviour” measure used in the current study. Although using a brief, accessible COM-B measure (Keyworth et al. 2020) is beneficial in terms of participant time, effort and response standardisation, it did not allow for an in-depth understanding of how, or whether, different types of hearing support, e.g. hearing aids vs. non-verbal communication techniques, are used by LTCH staff or the quality of hearing support provided. A more specific measure, e.g. relating only to the use of hearing aids, may have provided more reliable results. Future observational or qualitative studies may provide further insight into the target behaviour and could enhance the effectiveness of future interventions.

Although our sample encompassed staff from across the UK working in various LTCHs, it lacked ethnic diversity as most respondents were White British. These results are therefore not truly representative of UK-based LTCH staff, where 23% are from ethnic minority backgrounds (Skills for Care, 2022). In future, greater attention must be paid to ensure that staff from these communities are involved in research of this type, e.g. offering greater incentives and co-developing studies with LTCH staff from ethnic minority backgrounds (Farooqi et al. 2022).

## Conclusions

This study is a first step in the development of an evidence-based intervention to improve hearing support within LTCHs for residents with dementia. It provides insight into which LTCH staff and LTCH type are more likely to benefit from intervention, and in which COM domain. For the first time, this study identifies contextual issues and opportunities for the provision of hearing support using a well-established structural behaviour change framework (Michie, Van Stralen, and West 2011). The use of this model allows for a novel, theoretically driven, evidence-based intervention development that focuses on opportunity-based targets for intervention, in addition to staff knowledge and training.

## Supplementary Material

Supplemental Material
