# Supplementary material for: Capabilities, opportunities and motivations of staff to provide hearing support to long-term care home residents with dementia
Source: Int J Audiol. 2023 Jun 29;63(7):519–26. doi: 10.1080/14992027.2023.2227764 (PMC11225945; doi:10.1080/14992027.2023.2227764)
Supplement: Supplemental Material [file IIJA_A_2227764_SM9286.docx]

**Supplementary Materials**

**Survey:**

Thank you for taking the time to complete this questionnaire. The results will help to give us a better understanding of how you provide hearing loss support to residents with dementia and hearing loss. There are no right or wrong answers; all responses are **confidential** and **anonymous**.

Demographic information:

Gender:

- *Male*
- *Female*
- *Prefer not to answer*
- *Prefer to self-describe as:*

Age: [COMMENT BOX]

Ethnicity:

- *White*
- *Mixed/ multiple ethnic group*
- *Asian/ Asian British*
- *Back/ African/ Caribbean/ Black British*
- *Any other ethnic group*
- *Prefer not to answer*

Place of work:

- *Residential Home*
- *Nursing Home*
- *Dementia Specialist Home*
- *Don’t know*

Your place of work is owned by a:

- *Local authority*
- *Private company*
- *Don’t know*

Number of resident bedrooms in place of work:

[COMMENT BOX]

Job Title:

- *Facility Manager*
- *Registered Nurse (RGN/RNM)*
- *Senior Care Worker*
- *Care Worker (Care Assistant/ Support Worker/ Nursing Home Assistant)*
- *Other*
- *Prefer not to answer*

Qualifications (Please select your level of highest qualification):

- *Postgraduate qualification (Master’s or Doctorate)*
- *Undergraduate degree or equivalent*
- *Diploma, certificate or equivalent*
- *A-Level or equivalent*
- *GCSE or equivalent*
- *No qualifications*
- *Other*
- *Prefer not to answer*

Years in profession:

[COMMENT BOX]

The following questions are about the residents that you care for in a typical working week. We will then ask about how you provide **hearing loss support**. Hearing loss support includes: helping residents with their hearing aids or other hearing devices, using communication aides such as pictures or flashcards or changing your communication techniques to help those with hearing loss.

Out of the residents that you care for, how many have dementia?

| 0% |  | 100% |
| --- | --- | --- |

Out of the residents with dementia that you care for, how many do you think would benefit from hearing loss support?

| 0% |  | 100% |
| --- | --- | --- |

Out of the residents with dementia that you think would benefit, how many do you provide hearing loss support to?

| 0% |  | 100% |
| --- | --- | --- |

I am **physically** able to provide hearing loss support for residents with dementia

(For example: having the skills to insert hearing aids or change batteries)

*Strongly Disagree 0 1 2 3 4 5 6 7 8 9 10 Strongly Agree*

I am **psychologically** able to provide hearing loss support for residents with dementia

(For example: knowing and remembering who has hearing loss, knowing how to check that a hearing aid is working)

*Strongly Disagree 0 1 2 3 4 5 6 7 8 9 10 Strongly Agree*

Do you carry out testing or checking of hearing aids?

- *Yes*
- *No*

If so, how is this done:

[COMMENT BOX]

Providing hearing loss support for residents with dementia is something that I do **automatically**

(For example: it is part of your routine, you don’t think about it before doing it)

*Strongly Disagree 0 1 2 3 4 5 6 7 8 9 10 Strongly Agree*

I am **motivated** to provide hearing loss support to residents with dementia

(You have the desire to or feel the need to do this)

*Strongly Disagree 0 1 2 3 4 5 6 7 8 9 10 Strongly Agree*

Compared to other aspects of care (nutrition and hydration, skin integrity etc.), providing hearing loss support is a high priority for me

*Strongly Disagree 0 1 2 3 4 5 6 7 8 9 10 Strongly Agree*

Do you have specifically designated staff who are responsible for care of hearing

(For example putting a hearing aid in, changing batteries)?

- *Yes*
- *No*

Who is responsible for providing hearing loss support for residents with dementia?

(For example putting hearing aids in, changing the batteries)

- *Care Staff*
- *Nurses*
- *Relatives*
- *Resident*
- *Combined Effort/ Collaborative*
- *Other:* [Comment Box]

I have the **physical opportunity** to provide hearing loss support for residents with dementia

(For example: having enough time, having hearing aids provided)

*Strongly Disagree 0 1 2 3 4 5 6 7 8 9 10 Strongly Agree*

I have the **social opportunity** to provide hearing loss support for residents with dementia

(For example: staff working together, support from managers)

*Strongly Disagree 0 1 2 3 4 5 6 7 8 9 10 Strongly Agree*

Most residents with dementia who need a hearing aid (or other assistive hearing device) use one efficiently:

*Strongly Disagree 0 1 2 3 4 5 6 7 8 9 10 Strongly Agree*

If not, why?

- *Not Fitting Well*
- *Hard To Use*
- *Not Tolerated/ Refuses*
- *Too Expensive*
- *Lost Or Broken*
- *Not Effective*
- *Resident Forgets To Use Them*
- *Other (State):* [Comment Box]

I think that hearing loss support needs to be adapted for those who have dementia compared with those who do not have dementia:

*Strongly Disagree 0 1 2 3 4 5 6 7 8 9 10 Strongly Agree*

If so, why? What are the dementia-related reasons for this adaptation?

[COMMENT BOX]

The following questions are about the **training** that you receive on hearing loss support:

I have **training** and support to use sensory support equipment:

(For example how to use hearing aids, amplifiers, how to recognise if a resident has hearing loss)

- *Yes*
- *No*

If ‘yes’ please describe the training:

(For example was it compulsory, how many hours, did you receive a certificate)

[COMMENT BOX]

If ‘yes’, how recent was this?

- *Within The Last 12 Months*
- *Within The Last 5 Years*
- *Within The Last 10 Years*
- *I Have Not Had Training On This*

I would like additional training on how to support residents with hearing loss:

- *Yes*
- *No*

What do you think, if anything, could be improved about your training to better equip you to provide hearing loss support:

[COMMENT BOX]

**Supplementary Table.**

*Pearson product–moment correlation coefficients (two-tailed) for the target Behaviour and predictors entered into the regression model. Correlation coefficients in boldface indicate significant results (*p ≤ 0.05; **p ≤ 0.01).*

|  | 1 | 2 | 3 | 4 | 5 | 6 | 7 | 8 | 9 | 10 | 11 | 12 | 13 | 14 | 15 | 16 |
| --- | --- | --- | --- | --- | --- | --- | --- | --- | --- | --- | --- | --- | --- | --- | --- | --- |
| 1 Behaviour | - |  |  |  |  |  |  |  |  |  |  |  |  |  |  |  |
| 2 Nursing Home | -.06 | - |  |  |  |  |  |  |  |  |  |  |  |  |  |  |
| 3 Residential Home | .07 | **-.84**** | - |  |  |  |  |  |  |  |  |  |  |  |  |  |
| 4 Private Company | -.15 | .03 | .01 | - |  |  |  |  |  |  |  |  |  |  |  |  |
| 5 'Other' | .05 | **-.34**** | **.27**** | .11 | - |  |  |  |  |  |  |  |  |  |  |  |
| 6 Senior Carer | .09 | -.01 | .04 | .11 | -.12 | - |  |  |  |  |  |  |  |  |  |  |
| 7 Nurse | **.22**** | **.**09 | **-.18*** | .09 | -.12 | **-.22**** | - |  |  |  |  |  |  |  |  |  |
| 8 Manager | .04 | **-.28**** | **.24**** | -.13 | -.05 | -.09 | -.08 | - |  |  |  |  |  |  |  |  |
| 9 No. of bedrooms | -.14 | **.38**** | **-.34**** | **.28**** | -.12 | -.09 | -.15 | **-.18*** | - |  |  |  |  |  |  |  |
| 10 Years in profession | **-.19*** | **-.26**** | **.16*** | **.16*** | **.24**** | -.07 | -.10 | **.22**** | .07 | - |  |  |  |  |  |  |
| 11 Physical Capability | .08 | -.11 | .10 | **.17*** | .15 | **.41**** | .09 | -.01 | **-.33**** | -.03 | - |  |  |  |  |  |
| 12 Psychological Capability | **.23**** | **-.27**** | **.26**** | .02 | .12 | .15 | **.21**** | **.20*** | **-.49**** | -.06 | **.57**** | - |  |  |  |  |
| 13 Automatic Motivation | **.20*** | -.10 | .10 | **.20*** | .10 | **.39**** | -.11 | .15 | .02 | .05 | **.37**** | **.29**** | - |  |  |  |
| 14 Reflective Motivation | **.21*** | **-.30**** | **.20*** | .03 | **.18*** | **.23**** | **.19*** | **.18*** | **-.45**** | .02 | **.58**** | **.60**** | **.35**** | - |  |  |
| 15 Physical Opportunity | **.24**** | -.08 | .09 | **.25**** | .09 | **.25**** | .04 | .08 | -.09 | -.02 | **.45**** | **.50**** | **.46**** | **.44**** | - |  |
| 16 Social Opportunity | **.21**** | -.12 | .11 | **.15*** | **.19*** | .14 | **.23**** | .04 | **-.21**** | -.06 | **.39**** | **.51**** | **.33**** | **.52**** | **.64**** | - |
